# Asthma control after recovery from mild to moderate COVID-19: from Omicron BA.2 to XBB– from a cohort in a university hospital in Hong Kong

**DOI:** 10.1186/s12890-025-03543-x

**Published:** 2025-02-11

**Authors:** Wang Chun Kwok, Chung Ki Tsui, Terence Chi Chun Tam, David Chi Leung Lam, Mary Sau Man Ip, James Chung Man Ho

**Affiliations:** Department of Medicine, Queen Mary Hospital, The University of Hong Kong, 4/F, Professorial Block,102 Pokfulam Road, Pokfulam, Hong Kong Special Administrative Region China

**Keywords:** COVID-19, Asthma, Asthma control, Omicron, BA.2, XBB, Asthma control test

## Abstract

**Background:**

At the time of Omicron BA.2 outbreak, it was shown that mild to moderate COVID-19 was associated with worsening of asthma control after recovery. Whether the same phenomenon was also observed at a later phase of COVID-19 pandemic by other variants have not been reported.

**Methods:**

We conducted a follow-up study on patients with asthma who received clinical care in Queen Mary Hospital. The patients were first recruited in the study entitled “Worsening of asthma control after recovery from mild to moderate COVID-19 in patients from Hong Kong”. The primary outcome was the asthma control test (ACT) score difference among the patients who never had COVID-19 (no COVID-19 group), patients who had COVID-19 diagnosed in the initial study (past COVID-19 group) and patients who had COVID-19 diagnosed in the follow-up period (new COVID-19 group) of the current study.

**Results:**

189 patients were included. The change of ACT score from the last visit in the previous study to the last follow-up visit in current study was − 0.34 ± 3.7 in the no COVID-19 group, -0.0 ± 5.0 in the past COVID-19 group and − 0.17 ± 4.5 in the new COVID-19 group (*p* = 0.94). There were 10 (24.4%), 24 (25.5%) and 12 (22.2%) patients in the no COVID-19, past COVID-19 and new COVID-19 group who had worsening of asthma control by an increase in ACT score ≥ 3 from the last visit in the previous study to the last follow-up visit in current study (*p* = 0.90).

**Conclusion:**

Patients who had COVID-19 in 2023 with Omicron XBB as the dominant strain did not have worsening of asthma control seen in previous study done in 2022 with Omicron BA.2 as the circulating strain. Patients who had worsening of asthma control after COVID-19 in 2022 had subsequent improvement of asthma control with longer follow-up interval.

## Background

Since the outbreak of coronavirus disease 2019 (COVID-19) in year 2020, the virus evolves over time with different strains emerging. In the year 2021, Omicron variant was first discovered which subsequent became the dominant strain. Omicron sub-lineages with increasingly greater replication advantages emerged, replacing the previous predominant sub-lineage. The original Omicron variant was sub-lineage BA.1 and later followed by other sub-lineages. Several Omicron sub-lineages have a replication advantage [1] over the Delta variant and evade infection- and vaccine-induced humoral immunity to a greater extent than prior variants [2]. They also appear to be associated with less severe disease than other variants [3].

The impact of COVID-19 on asthma has been demonstrated in our previous study conducted in 2022, with patients recruited from 24th May 2022 to 1st November 2022. In that study, recovery from mild-to-moderate COVID-19 in the last 30 to 270 days among asthma patients was shown to be associated with worsening of asthma symptom, lower ACT score, a higher need for escalation of asthma maintenance therapy and more uncontrolled asthma [4]. Omicron BA.2 was the dominant variant during the study period [5]. In 2023, XBB variant gradually replaced BA.2 and became the dominant variant [6].

Whether the same phenomenon was observed among different Omicron strains, and whether the patients who had initial asthma worsening subsequently improved were important clinical questions. As such, we conducted this study to assess the association between different Omicron strains and the impact on asthma control, as well as follow-up on those who had initial worsening of asthma control.

## Methods

### Study design and data sources

This is a follow-up study of our previously published investigation entitled “Worsening of asthma control after recovery from mild to moderate COVID-19 in patients from Hong Kong” [4]. Patients recruited from 24th May 2022 to 1st November 2022 were followed up till 30th September 2023. In the initial study, there were 221 adult patients with asthma, among which 111 (50.0%) had COVID-19, at the time Omicron BA.2 being the dominant variant. To be consistent with our prior published study, patients with severe COVID-19 were excluded. The detailed methodology of the initial study can be found in Kwok et al. [4]All patients were followed up from 2nd November 2022 till 30th September 2023, in which the dominant strain was gradually replaced by Omicron XBB. Their asthma control was assessed by asthma control test (ACT). ACT was assessed for these patients at the following time points: [1] Enrolment visit [2] 6 months after enrollment visit [3] 12 months after enrollment visit. The number of asthma exacerbation, as well as COVID-19 reinfections and their severity in both groups during the follow-up period were recorded. The severity of asthma was defined using National Institutes of Health criteria [7]. COVID-19 was diagnosed and confirmed by laboratory-confirmed positive reverse transcription–polymerase chain reaction (RT-PCR) test, or positive rapid antigen test (RAT), as documented on the designated COVID-19 data platform on Clinical Management System (CMS) of Hong Kong Hospital Authority (HKHA). Patients’ records were accessed through the electronic patient record (ePR) of the HKHA. Patient demographics (age, sex, smoking status), clinical records (ACT score [8], asthma medication, comorbidities, spirometry results, date of COVID-19, hospitalization and complications from COVID-19, date and dose of COVID-19 vaccination, type of COVID-19 vaccines), investigation results and treatment records were retrieved from ePR and clinical records. The past ACT score, asthma medication, comorbidities, spirometry results and COVID-19 vaccination details were collected from a retrospective database. Spirometry was performed with CareFusion Vmax^®^ Encore 22 system. Spirometry was done within 12 months of the recruitment visit. Spirometry data was interpreted with the updated spirometric reference values for adult Chinese in Hong Kong [9]. The study was approved by the Institutional Review Board of the University of Hong Kong and Hospital Authority Hong Kong West Cluster (UW 21–172). Informed consent was obtained from all participants. For the accordance statement, all methods were carried out in accordance with relevant guidelines and regulations.

The patients were separated into three different groups: No COVID-19 group (Never had COVID-19), past COVID-19 group (COVID-19 diagnosed in the initial study) and new COVID-19 group (COVID-19 diagnosed in the follow-up period of the current study).

### Outcomes

The primary outcome was the change in ACT score from the last visit in the prior study to the last visit in this follow-up study in patients among these three groups. The secondary outcome was the number of asthma exacerbations in the follow-up period, which are further stratified in the severity as those required out-patient management with oral corticosteroid, those required hospitalization and those required intensive care unit admission or mechanical ventilation. The need for escalation of asthma treatment by increase in at least 1 GINA step was also included as the secondary outcome.

### Statistical analysis

The demographic and clinical data were described in actual frequency, mean ± standard deviation (SD) or median (interquartile range). Baseline demographic and clinical data were compared between the three groups by Chi-squared test or Fisher’s exact test as appropriate. The continuous variables were compared between the three groups by one-way ANOVA. The risks of worsening asthma control between different patient groups were compared by binary logistic regression. Multiple logistic regression modeling was used to account for potential confounders including age, gender, smoking status, baseline forced expiratory volume in 1 second (FEV_1_ ) [% predicted], COVID-19 vaccination status, ACT score at prior visit 12 months before and GINA step of medication at baseline. The statistical significance was determined at the level of *p* < 0.05. All statistical analyses were done using the 28th version of SPSS statistical package.

## Results

**Fig. 1:**
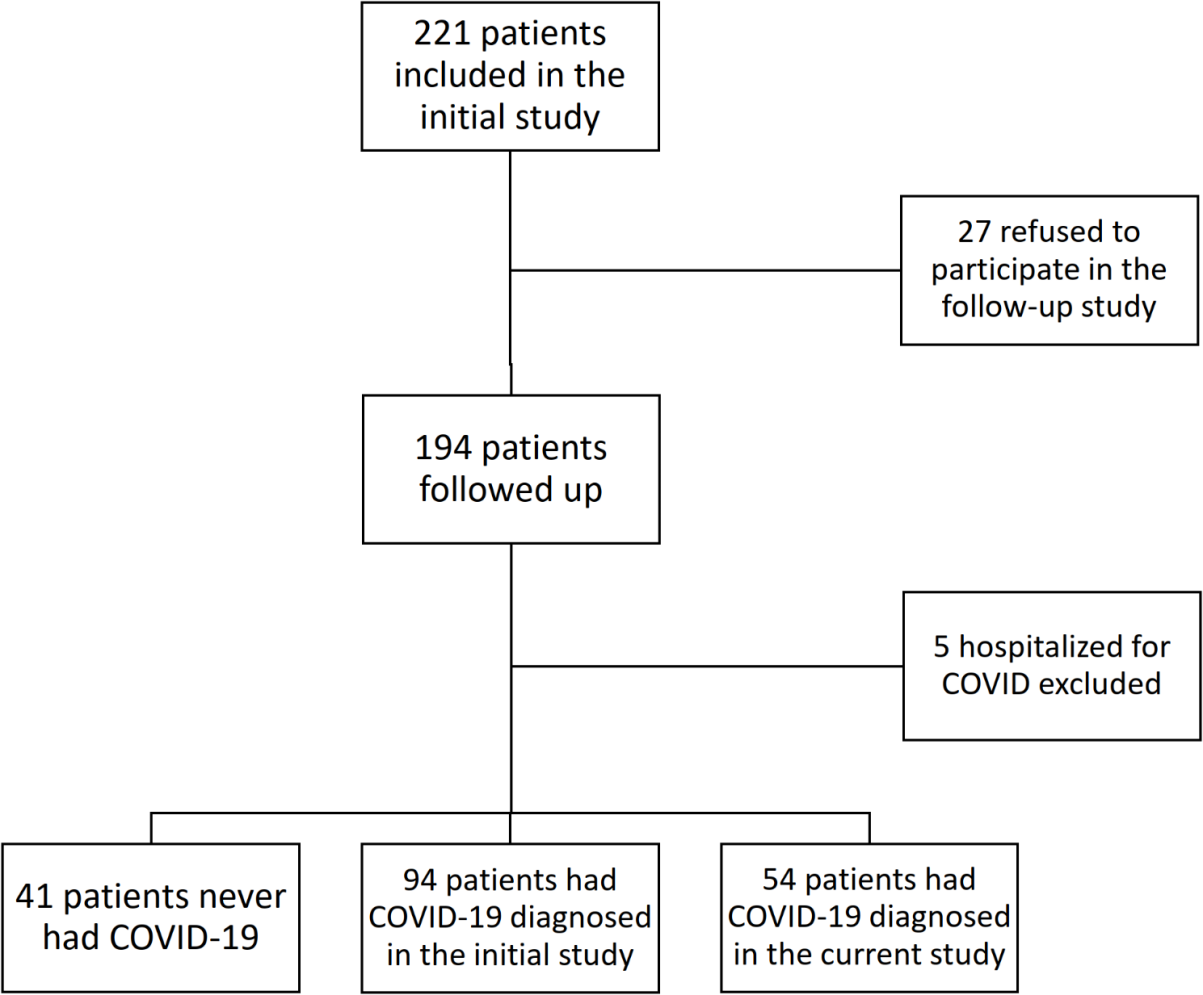
Patient selection flow diagram

Among the 221 adult patients with asthma recruited in the initial study, 27 refused to participate in the follow-up study. Among the 194 patients who were followed up, 59 had COVID-19 in the follow-up period and 5 required hospitalizations for COVID-19. This is illustrated in Fig. 1. After excluding the 5 patients hospitalized with COVID-19, 189 patients were included with mean age of 57.7 ± 15.4 years. There were 70 (37.0%) male patients and 154 (81.4%) were never smoker. Co-existing rhinosinusitis and atopic dermatitis were identified in 154 (81.4%) and 78 (41.3%) patients respectively. The mean baseline FEV_1_ was 2.10 ± 0.79 L (89.5 ± 23.1% predicted), with the baseline FEV_1_/FVC ratio was 67.3 ± 14.4% (Table 1). The mean ACT score as in the last visit of the previous study was 19.0 ± 4.9. In the first follow-up visit 6 months after the end of initial study, the mean ACT score was 19.9 ± 4.2, while it remained as 19.9 ± 4.2 at the last follow-up visit.

**Table 1:** Baseline demographic and clinical characteristics of included patients

	No COVID-19 (*n* = 41)	Past COVID-19 (*n* = 94)	New COVID-19 (*n* = 54)	Whole cohort (*n* = 189)	*p*-values^
Age (years), mean ± SD	59.9 ± 15.5	56.3 ± 15.7	58.6 ± 14.7	57.7 ± 15.4	0.398
Gender					0.462
Male	18 (43.9%)	35 (37.2%)	17 (31.5%)	70(37.0%)	
Female	23 (56.1%)	59 (62.8%)	37 (68.5%)	119 (63.0%)	
Smoking status					0.799
Non-smoker	34 (82.9%)	76 (80.9%)	44 (81.5%)	154 (81.5%)	
Active smoker	2 (4.9%)	9 (9.6%)	6 (11.1%)	17 (9.0%)	
Former smoker	5 (12.2%)	9 (9.6%)	4 (7.4%)	18 (9.5%)	
Co-morbidities					
Rhinosinusitis	28 (68.3%)	82 (87.2%)	44 (81.5%)	154 (81.5%)	0.152
Atopic dermatitis	18 (43.9%)	37 (39.4%)	23 (42.6%)	78 (41.3%)	0.862
GINA steps					0.402
1	1 (2.4%)	2 (2.1%)	0 (0%)	3 (1.6%)	
2	6 (14.6%)	12 (12.8%)	2 (3.7%)	20 (10.6%)	
3	14 (34.1%)	31 (33.0%)	22 (40.7%)	67 (35.4%)	
4	12 (29.3%)	29 (30.9%)	23 (42.6%)	64 (33.9%)	
5	8 (19.5%)	20 (21.3%)	7 (13.0%)	35 (18.5%)	
COVID vaccination status					0.18
Incomplete	5 (12.2%)	12 (12.8%)	2 (3.7%)	19 (10.1%)	
Completed 2 doses	36 (87.8%)	82 (87.2%)	52 (96.3%)	170 (89.9%)	
Asthma treatment					
As needed short acting bronchodilator	1 (2.4%)	2 (2.1%)	0 (0%)	3 (1.6%)	0.40
Regular inhaled corticosteroid only	6 (14.6%)	12 (12.8%)	2 (3.7%)	20 (10.6%)	0.28
Long acting bronchodilator with low dose inhaled corticosteroid	14 (34.1%)	31 (33.0%)	22 (40.7%)	67 (35.4%)	0.44
Long acting bronchodilator with medium dose inhaled corticosteroid	12 (29.3%)	29 (30.9%)	23 (42.6%)	64 (33.9%)	0.51
Long acting bronchodilator with medium/high dose inhaled corticosteroid and long acting anti-muscarinic	8 (19.5%)	20 (21.3%)	7 (13.0%)	35 (18.5%)	0.28
Montelukast	20 (48.8%)	47 (50.0%)	23 (42.6%)	90 (47.6%)	0.68
Theophylline	4 (9.8%)	17 (8.1%)	9 (16.7%)	30 (15.9%)	0.47
Biologics	0 (0%)	0 (0%)	1 (1.9%)	1 (0.5%)	0.29
Maintenance systemic corticosteroid	0 (0%)	0 (0%)	0 (0%)	0 (0%)	-
Baseline FEV_1_ (L), mean ± SD	2.16 ± 0.89	2.12 ± 0.84	2.05 ± 0.62	2.11 ± 0.79	0.816
Baseline FEV_1_ (% predicted), mean ± SD	89.2 ± 24.6	88.7 ± 21.6	91.2 ± 24.7	89.5 ± 23.1	0.855
Baseline FVC (L), mean ± SD	3.27 ± 0.93	3.10 ± 1.00	3.13 ± 0.97	3.13 ± 0.97	0.769
Baseline FVC (% predicted), mean ± SD	107.1 ± 16.7	103.4 ± 18.4	127.1 ± 139.6	111.4 ± 76.3	0.272
Baseline FEV_1_ to FVC ratio, mean ± SD	66.0 ± 15.9	67.9 ± 14.2	67.1 ± 13.6	67.3 ± 14.4	0.809
Baseline eosinophil count (x cells/µL), mean ± SD	302 ± 289	320 ± 264	249 ± 195	296 ± 253	0.262
ACT at enrolment visit, mean ± SD	20.5 ± 4.0	17.5 ± 4.5	20.5 ± 3.9	19.0 ± 4.9	< 0.001
ACT score at the first follow-up visit 6 months, mean ± SD	20.0 ± 3.7	19.8 ± 4.3	20.0 ± 4.4	19.9 ± 4.2	0.968
ACT score at the follow-up visit 12 months, mean ± SD	19.9 ± 4.2	19.7 ± 4.1	20.1 ± 4.3	19.9 ± 4.2	0.903
Asthma control by ACT score in the last visit in the previous study					0.107
Controlled	21 (51.2%)	45 (47.9%)	28 (51.9%)	94 (49.7%)	
Partially controlled	17 (41.5%)	28 (29.8%)	21 (38.9%)	66 (34.9%)	
Uncontrolled	3 (7.3%)	21 (22.3%)	5 (9.3%)	29 (15.3%)	
Asthma control by ACT score at the first follow-up visit 6 months					0.840
Controlled	26 (63.4%)	56 (59.6%)	34 (63.0%)	116 (61.4%)	
Partially controlled	9 (22.0%)	18 (19.1%)	12 (22.2%)	39 (20.6%)	
Uncontrolled	6 (14.6%)	20 (21.3%)	8 (14.8%)	34 (18.0%)	
Asthma control by ACT score at the follow-up visit 12 months					0.903
Controlled	20 (48.8%)	43 (45.7%)	29 (53.7%)	92 (48.7%)	
Partially controlled	10 (24.4%)	27 (28.7%)	13 (24.1%)	50 (26.5%)	
Uncontrolled	11 (26.8%)	24 (25.5%)	12 (22.2%)	47 (24.9%)	

*: statistically significant; SD: standard deviation; IQR: Interquartile range; µL: microliters; L: Liter; ACT: asthma control test; FEV_1_: forced expiratory volume in one second; FVC: forced vital capacity; GINA: Global Initiative for Asthma

### ACT score

**Fig. 2:**
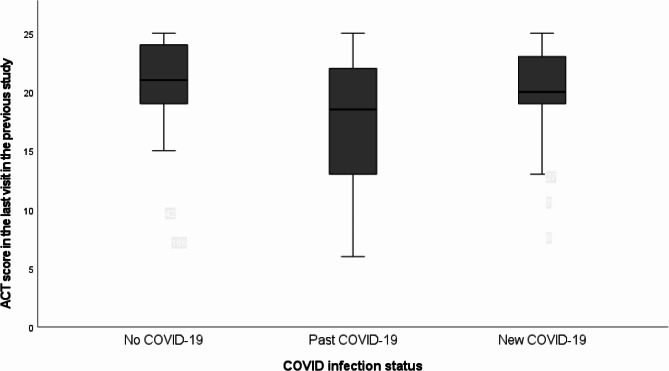
ACT score in the last visit in the previous study among the three groups

The ACT score at the last visit in the previous study was 20.5 ± 4.0 in the no COVID-19 group, 17.5 ± 5.4 in the past COVID-19 group and 20.5 ± 3.9 in the new COVID-19 group (*p* < 0.001 in univariate analysis, *p* = 0.033 in multi-variate analysis) (Fig. 2).

**Fig. 3:**
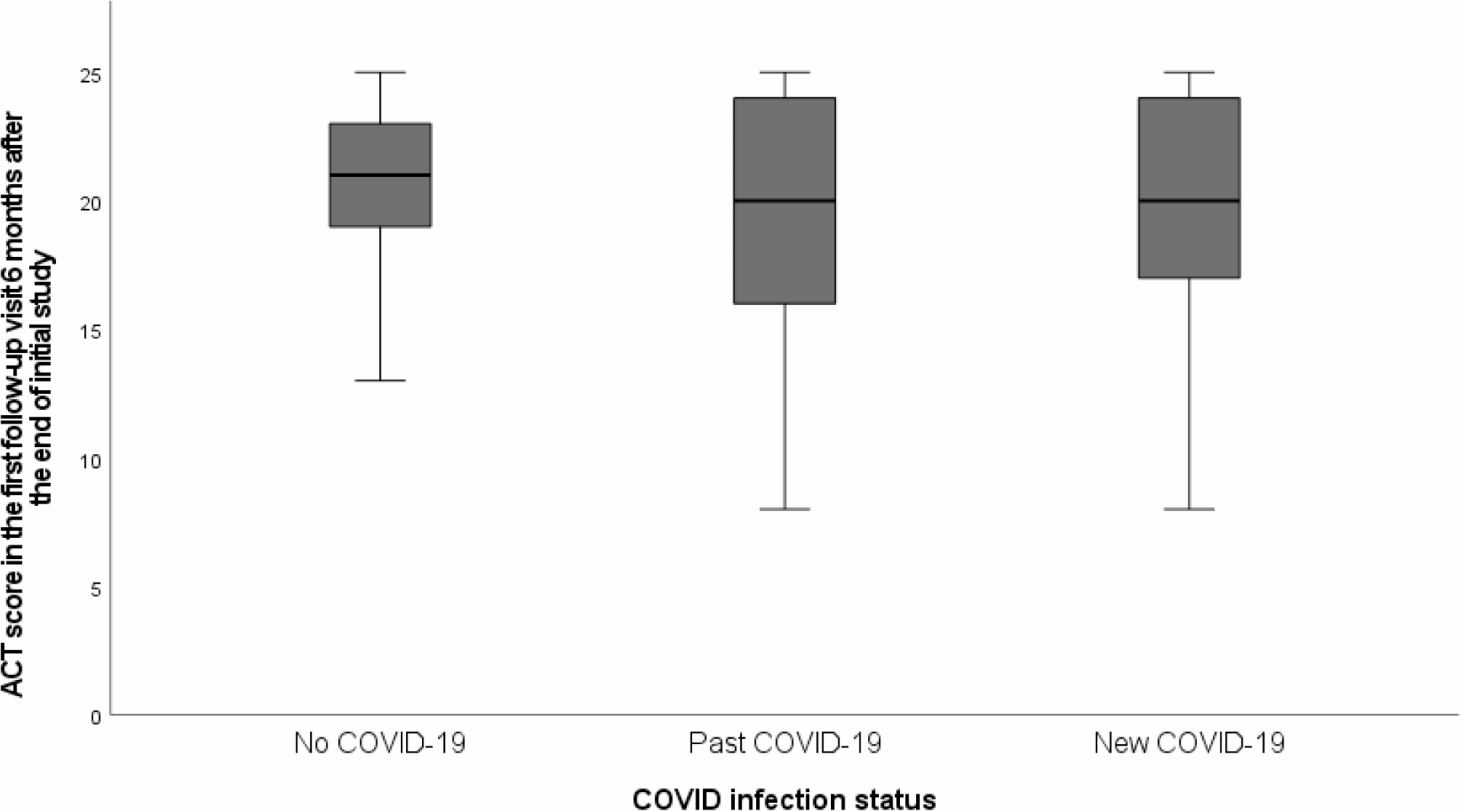
ACT score in the first follow-up visit 6 months after the end of initial study among the three groups

The ACT score at the first follow-up visit 6 months after the end of initial study was 20.0 ± 3.7, 19.8 ± 4.3 and 20.0 ± 4.4 in the no COVID-19, past COVID-19 and new COVID-19 group respective (*p* = 0.97, in univariate analysis, *p* = 0.60 in multi-variate analysis) (Fig. 3).

**Fig. 4:**
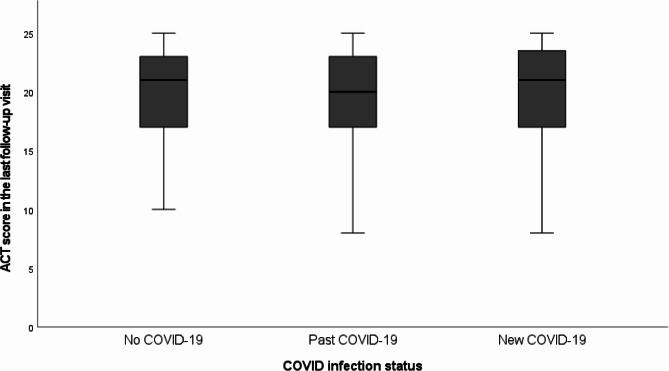
ACT score in the last follow-up visit

Similarly, the ACT score at the last follow-up visit in current study was 19.9 ± 4.2, 19.7 ± 4.1 and 20.0 ± 4.3 in the no COVID-19, past COVID-19 and new COVID-19 group respectively (*p* = 0.90, in univariate analysis, *p* = 0.75 in multi-variate analysis) (Fig. 4).

The change of ACT score from the last visit in the previous study to the last follow-up visit in current study was − 0.34 ± 3.7 in the no COVID-19 group, -0.0 ± 5.0 in the past COVID-19 group and − 0.17 ± 4.5 in the new COVID-19 group (*p* = 0.94 in univariate analysis, *p* = 0.70 in multi-variate analysis).

There were 10 (24.4%), 24 (25.5%) and 12 (22.2%) patients in the no COVID-19 group, past COVID-19 group and new COVID-19 group who had worsening of asthma control by increase in ACT score ≥ 3 from the last visit in the previous study to the last follow-up visit in current study (*p* = 0.90).

### Change in asthma treatment

3 (7.3%), 15 (16.0%) and 2 (3.7%) of the patients in the no COVID-19 group, past COVID-19 group and new COVID-19 group who had increase in asthma medication by increase in at least 1 GINA step. Using the no COVID-19 group as reference group, the odd ratios (OR) were 2.41 (95% confidence interval [CI] = 0.66–8.81, *p* = 0.19) for the past COVID-19 and 0.49 (95% CI = 0.08–3.06, *p* = 0.44) for the new COVID-19 group. The adjusted OR (aOR) were 3.36 (95% CI 0.59–19.13, *p* = 0.17) for the past COVID-19 and 1.58 (95% CI = 0.18–14.26, *p* = 0.69) for the new COVID-19 group.

### Asthma exacerbation

There were no patients in the no COVID-19 group having all asthma exacerbation in the follow-up period, while there were 8 (8.5%) and 2 (3.7%) of the patients in past COVID-19 group and new COVID-19 group to have all asthma exacerbation in the follow-up period (*p* = 0.11). Among these exacerbations, all the exacerbations in the new COVID-19 group were managed in out-patient setting with oral corticosteroid. Among the patients in the past COVID-19 group, 6 of them had exacerbations managed in out-patient setting with oral corticosteroid and 2 required hospitalization.

There were no patients in the no COVID-19 and new COVID-19 groups having hospitalized asthma exacerbation in the follow-up period, while there were 2 (2.1%) in past COVID-19 group to have hospitalized asthma exacerbation in the follow-up period (*p* = 0.36).

There was no asthma exacerbation that required intensive care unit admission or mechanical ventilation in the 3 patient groups.

## Discussion

Our follow-up study suggested the possibility that different variants of SARS-CoV-2 might have different impact on asthma control after mild COVID-19. Unlike what our group showed in prior study, those infected in 2023 with XBB being the dominant variant were less likely to have worsening of asthma control. The result is consistent with the disease severity of COVID-19 due to different variants [10].

While our prior study suggested that there was worsening of asthma control after recovery of mild-to-moderate COVID-19, the same observation was not seen in the current study. One postulation is that the variant of SARS-CoV-2 may play a role. Different variants of SARS-CoV-2 may have different virulence, leading to different severity of COVID-19. This may also affect the aftermath of COVID-19. With a virus invading the respiratory system, the more severe the disease, the more pronounced local consequences after recovery from the infection will be anticipated. The consequences observed in the prior study could reflect the local damage to the airway epithelium from SARS-CoV-2, which resulted in worsening of asthma control even after the recovery from COVID-19. These patients eventually have improved asthma control in the longer follow-up period as demonstrated in this study, with ACT score gradually increased to the level close to those without COVID-19 infection. While for those who had COVID-19 diagnosed in 2023, they did not have significant changes in ACT score throughout the follow-up period. In the prior study and the current study, all the included cases had mild to moderate COVID-19 that did not require hospitalization. Any observed clinical consequences are unlikely due to severe COVID-19. Other possible explanation of this phenomenon includes the increase in vaccination rate after the fifth wave of COVID-19 which overwhelmed the public health care system in Hong Kong and raised the public awareness of COVID-19 vaccination. The availability of anti-viral may also play a part. Herd immunity after the major outbreak in early 2022 could be another explanation.

Since the start of COVID-19 pandemic, there are many researches on the association of COVID-19 and asthma, which happen across different time points, having different strains of SARS-CoV-2 and with the vaccines and anti-viral available subsequently. It is not surprised that the viral strains, vaccines and anti-viral would have impact on the association between COVID-19 and asthma control. Recently, there are evidence that the risks of outcome among asthma patients with COVID-19 actually differ in different subgroups. In a population-based cohort of asthma patients from the Swedish National Airway Register (SNAR), they identified distinct differences in COVID-19-related risk factors among asthma patients of different ages [11]. The impact of COVID-19 on asthma at different time point, indicating the impact from various strains SARS-CoV-2 with different pathogenicity may also differ.

A hot topic in the COVID-19 related research is the persistent consequences from COVID-19. Post-COVID syndrome or long-COVID is a syndrome encompassing a protracted course of various physical and neuropsychiatric symptoms that persist for more than 12 weeks after COVID-19 without an alternative explanation [12–16]. While post-COVID syndrome or long-COVID reflects a group of more general symptoms after recovery from COVID-19, as a respiratory tract infection, patients may experience more local damage from COVID-19. In patients with asthma, it could manifest as worsening of symptoms, which is objectively reflected by ACT score. Some may even experience asthma exacerbation. Whether the viral strain also affects the prevalence and severity of post-COVID syndrome also warrants further research. Nonetheless, the importance of protection against COVID-19 with vaccination and early treatment with anti-viral for patients with asthma should not be over-emphasized, as different variants are emerging with time. The phenomenon observed in this study shall not be a false reassurance to patients with asthma, especially when other respiratory pathogens are also returning which could also contribute to worsening of asthma with asthma exacerbations.

There are a few limitations in our study. First, this study involved only a single centre. However, the respiratory unit in our tertiary medical centre received referrals from all other healthcare sources, and patients diagnosed with asthma were managed in a designated asthma clinic, and the patients in this study had comprehensive clinical data including lung function test results. Secondly, the patients were diagnosed to have COVID-19 by RAT or PCR, and therefore the measurement of viral load at the time of infection was not possible. Instead, in this study, clinical severity of COVID-19 were used to gauge the severity of disease, which is also adopted in other studies. Thirdly, ACT was used as the primary outcome, as we did in the initial study [4]. This can assess patients’ asthma control mainly by their symptoms. Repeat lung function and fractional exhalation of nitric oxide (FeNO) which reflects airway eosinophilic inflammation was not monitored in the current study as these were not within the protocol of the initial published study [4]. It will be ideal if the assessment could include physiological measures such as FEV_1_ and inflammatory parameters with FeNO. Another limitation is that other viral or bacterial infections could also lead to changes in asthma control. While all the patients in this cohort did not have co-existing or concurrent viral or bacterial infections in the study period, in the post-COVID era, the resurgence of various viral and bacterial infections could complicate the scenario, which may affect the application of this research finding in the real-world situation in the future [17].

## Conclusion

Patients who had COVID-19 in 2023 with Omicron XBB being dominant strain did not have worsening of asthma control as observed in the previous study done in 2022 which mainly included the Omicron BA.2 strain. Patients who had worsening of asthma control after COVID-19 in 2022 had subsequent improvement of asthma control in longer follow-up interval.

## Data Availability

The datasets supporting the conclusions of this article are included within the article and no additional data will be provided.

## Abbreviations

ACT: Asthma control test
aOR: Adjusted odds ratio
CI: Confidence interval
CMS: Clinical management system
COPD: Chronic obstructive pulmonary disease
COVID-19: Coronavirus disease 2019
ePR: Electronic patient record
FEV_1_: Forced expiratory volume in one second
FVC: Forced vital capacity
FeNO: Fractional exhalation of nitric oxide
GINA: Global initiative for asthma
IgE: Immunoglobulin E
IQR: Interquartile range
MID: Minimally important difference
OR: Odds ratio
RAT: Rapid antigen test
RT-PCR: Reverse transcription–polymerase chain reaction
SD: Standard deviation
